# Supplementary material for: Intersectional inequity in knowledge, attitude, and testing related to HIV in Ethiopia: People with multiple disadvantages are left behind
Source: PLOS Glob Public Health. 2024 Aug 22;4(8):e0003628. doi: 10.1371/journal.pgph.0003628 (PMC11340895; doi:10.1371/journal.pgph.0003628)
Supplement: S1 Table — (PDF) [file pgph.0003628.s001.pdf]

Multilevel mixed effect logistic regression analysis of inequity in comprehensive knowledge about HIV/AIDS among adults (both sexes) in Ethiopia, 2016 (N=27, 261)

Variables	Model 0 (Null model)	Individual level variables Model I aOR (95% CI)	Community level variables aOR (95% CI) (Modell II)	Model III aOR (95% CI)
**Age (reference: 15-19 years)**				
20-24		0.94 (0.85, 1.04)		1.10 (0.94, 1.28)
25-29		0.90 (0.81, 1.01)		1.03 (0.88, 1.21)
30-34		0.92 (0.81, 1.04)		1.15 (0.97, 1.37)
35-39		0.88 (0.77, 1.003)		1.19 (0.99, 1.44)
40-44		0.78 (0.68, 0.90)**		0.91 (0.74, 1.12)
45-49		0.79 (0.67, 0.92)**		1.10 (0.88, 1.37)
**Marital status (reference: married)**				
Never married		1.26 (1.15, 1.39)***		1.21 (1.07, 1.38)**
Widowed/divorced/no longer living together/separated		1.04 (0.92, 1.19)		0.94 (0.79, 1.12)
**Religion (reference: Orthodox)**				
Catholic		0.69 (0.47, 1.01)		0.74 (0.44, 1.25)
Protestant		0.84 (0.75, 0.93)**		0.91 (0.73, 1.15)
Muslim		0.69 (0.63, 0.76***		1.04 (0.91, 1.19)
Others		0.67 (0.46, 0.98)*		0.74 (0.41, 1.33)
**Sex of household head (reference: male)**				
Female		1.10 (1.01, 1.18)*		1.13 (1.003, 1.27)
**Reading newspaper (reference: No)**				
Yes		1.50 (1.39, 1.62)***		1.23 (1.10, 1.34)***
**Listening to radio (reference: No)**				
Yes		1.21 (1.12, 1.30)***		1.03 (0.92, 1.15)
**Watching television (reference: No)**				
Yes		1.58 (1.45, 1.71)***		1.45 (1.27, 1.67)***
**Ever been tested for HIV (reference: No)**				
Yes		1.80 (1.69, 1.94)***		1.38 (1.25, 1.55)***
**Marginalisation unemployed & female gender (sex & employment): Reference: unemployed female**				
Unemployed male		1.85 (1.61, 2.13)***		1.39 (1.08, 1.78)*
Employed female		1.16 (1.06, 1.27)*		1.12 (0.98, 1.28)
Employed male		2.46(2.27, 2.67)***		2.43 (2.07, 2.85)***
**Region (reference: Addis Ababa)**				
Tigray			1.17 (0.96, 1.43)	1.25 (1.01, 1.55)*

Afar			0.71 (0.53, 0.95)*	0.75 (0.55, 1.03)
Amhara			1.29 (1.06, 1.57)*	1.35 (1.10, 1.65)**
Oromia			0.87 (0.71, 1.07)	0.99 (0.80, 1.23)
Somali			0.22 (0.16, 0.29)***	0.28 (0.20, 0.37)***
Benshangul-Gumuz			0.66 (0.52, 0.84)**	0.76 (0.60, 0.97)*
SNNPR			0.86 (0.67, 1.07)	1.08 (0.84, 1.38)
Gambela			0.80 (0.65, 0.99)*	0.92 (0.73, 1.16)
Harari			0.60 (0.45, 0.80)**	0.66 (0.49, 0.89)**
Dire Dawa			0.69 (0.53, 0.91)**	0.66 (0.49, 0.90)*
**Marginalisation (wealth index, education, residence) reference:** Poor uneducated rural (PUR)				
Poor uneducated urban (PUU)			2.1 (1.06, 4.17)	1.64 (0.83, 3.27)
Poor educated rural (PER)			2.47 (2.15, 2.84)***	1.75 (1.50, 2.05)***
Poor educated urban (PEU)			4.87 (2.38, 9.97)***	3.16 (1.39, 7.18)**
Rich uneducated rural (RUR)			1.06 (0.89, 1.27)	1.00(0.83, 1.21)
Rich educated rural (RER)			3.01 (2.61, 3.47)***	2.04 (1.74, 2.40)***
Rich uneducated urban (RUU)			1.62 (1.17, 2.24)**	1.27 (0.90, 1.79)
Rich educated urban (REU)			5.91 (4.97, 7.04)***	3.41 (2.76, 4.21)***
**General contextual effects (random effects)**				
Variance (SE)	0.013 (1.3%)	0.009 (0.9%)	0.008 (0.8%)	0.008 (0.8%)
ICC %	0.137 (13.7%)	0.097 (9.7%)	0.061 (6.1%)	0.063 (6.3%)
Model fit statistics				
AIC	30754.45	27842.24	29479.79	28227.46
BIC	30770.88	28022.93	29635.85	28547.78
Log likelihood				-14074.73
**Standard logistic regression to compare with the final model**				
AIC				28829.06
BIC				29141.16
Log likelihood				-14376.53

AIC= Akaike's information criterion; aOR = adjusted odds ratio; BIC= Bayesian information criterion; ICC=intraclass correlation

Multilevel mixed effect logistic regression analysis of inequity in accepting attitudes towards people living with HIV among both sexes in Ethiopia, 2016 (N=25,542)

Variables	Model 0 (Null model)	Model-I [Individual level variables aOR (95% CI)]	Model-II [Community level variables aOR (95%CI)]	Model-III [individual and community level variables aOR (95% CI)]
**Age (reference: 15-19 years)**				
20-24		1.09 (0.99, 1.21)		1.27 (1.10, 1.48)**
25-29		1.04 (0.93,1.17)		1.27 (1.06, 1.52)*
30-34		1.00 (0.88, 1.14)		1.29 (1.07, 1.56)**

35-39		1.00 (0.88, 1.14)		1.26 (1.03, 1.54)*
40-44		1.00 (0.87, 1.15)		1.17 (0.95, 1.45)
45-49		0.93 (0.80 (1.09)		1.04 (0.82, 1.33)
**Marital status (reference: married)**				
Never married		1.61 (1.46, 1.77)***		1.44 (1.25, 1.66)***
Widowed/divorced/no longer living together/separated		1.14 (1.01, 1.29)*		1.10 (0.91, 1.34)
**Religion (reference: Orthodox)**				
Catholic		0.65 (0.44, 0.96)*		1.08 (0.59, 1.96)
Protestant		0.74 (0.66, 0.84)***		0.91 (0.74, 1.11)
Muslim		0.68 (0.61, 0.76)***		0.91 (0.76, 1.09)
Others		0.34 (0.22, 0.53)***		0.39 (0.17, 0.88)*
**Sex of household head (reference: male)**				
Female		1.10 (1.02, 1.19)*		1.16 (1.03, 1.31)*
**Reading newspaper (reference: No)**				
Yes		1.74 (1.61, 1.89)***		1.62 (1.41, 1.85)***
**Listening to radio (reference: No)**				
Yes		1.10 (1.03, 1.19)**		1.06 (0.89, 1.14)
**Watching television (reference: No)**				
Yes		1.55 (1.43, 1.68)***		1.26 (1.11, 1.44)***
**Ever been tested for HIV (reference: No)**				
Yes		1.76 (1.64, 1.88)***		1.34 (1.21, 1.49)***
**Sex and employment (unemployed female)**				
Unemployed male		1.10 (0.95, 1.23)		1.72 (1.34, 2.22)***
Employed female		1.09 (1.003, 1.19)*		1.01 (0.86, 1.19)
Employed male		1.09 (1.002, 1.18)*		1.25 (0.99, 1.58)
**Comprehensive knowledge** (reference: No)				
Yes		2.26 (2.11, 2.42)***		2.06 (1.85, 2.31)***
**Region (reference: Addis Ababa)**				
Tigray			0.78 (0.58, 1.04)	0.76 (0.57, 1.04)
Afar			0.82 (0.61, 1.10)	0.99 (0.71, 1.39)
Amhara			0.93 (0.72, 1.20)	1.00 (0.77, 1.31)
Oromia			0.44 (0.33, 0.61)***	0.55 (0.40, 0.76)***
Somali			0.20 (0.15, 0.26)***	0.29 (0.21, 0.40)***
Benshangul-Gumuz			0.69 (0.52, 0.93)*	0.92 (0.68, 1.24)
SNNPR			0.41 (0.30, 0.54)***	0.50 (0.38, 0.68)***
Gambela			0.78 (0.57, 1.07)	0.96 (0.69, 1.32)
Harari			0.74 (0.58, 0.96)*	0.97 (0.74, 1.27)
Dire Dawa			0.78 (0.61, 1.00)	0.96 (0.74, 1.23)
**Marginalisation (wealth index, education, residence) reference:** Poor uneducated rural (PUR)				
Poor uneducated urban (PUU)			2.20 (0.94, 5.17)	1.65 (0.70, 3.87)

Poor educated rural (PER)			2.96 (2.43, 3.60)***	2.07 (1.68, 2.54)***
Poor educated urban (PEU)			4.89 (2.42, 9.90)***	2.51 (1.23, 5.12)***
Rich uneducated rural (RUR)			1.38 (1.15, 1.66)**	1.35 (1.12, 1.64)**
Rich educated rural (RER)			4.00 (3.32, 4.83)***	2.56 (2.11, 3.11)***
Rich uneducated urban (RUU)			4.58 (3.36, 6.24)***	3.60 (2.66, 4.88)***
Rich educated urban (REU)			15.50 (12.24, 19.63)***	7.3 (5.79, 9.24)***
Variance (SE)	0.014 (1.4%)	0.011 (1.1%)	0.009 (0.9%)	0.009 (0.9%)
ICC %	0.291 (29.1%)	0.179 (17.9%)	0.110 (11.0%)	0.109 (10.9%)
Model fit statistics				
AIC	29901.56	28026.34	28191.63	27037.82
BIC	29917.86	28213.74	28346.44	27363.75
Log likelihood				-13478.91
Standard logistic regression				
AIC				28003.16
BIC				28320.93
Log likelihood				-13962.58

Multilevel mixed effect logistic regression analysis of inequity in recent HIV test among adults (both sexes) in Ethiopia, 2016 (N=25,542)

Variables	Model 0 (Null model)	Model I aOR (95% CI)	Model II aOR (95% CI)	Model III aOR (95% CI)
**Age (reference: 15-19 years)**				
20-24		2.09 (1.88, 2.34)***		2.19 (1.81, 2.64)***
25-29		2.19 (1.94, 2.47)***		2.20 (1.76, 2.74)***
30-34		1.71 (1.50, 1.95)***		1.84 (1.45, 2.35)***
35-39		1.42 (1.23, 1.63)***		1.41 (1.11, 1.79)**
40-44		1.19 (1.02, 1.39)*		1.43 (1.09, 1.88)**
45-49		0.99 (0.84, 1.19)		1.05 (0.77, 1.43)
**Marital status (reference: married)**				
Never married		0.56 (0.50, 0.61)***		0.48 (0.41, 0.56)***
Widowed/divorced/no longer living together/separated		0.86 (0.78, 0.98)*		0.86 (0.69, 1.06)
**Religion** (reference: Orthodox)				
Catholic		0.93 (0.62, 1.40)		0.62 (0.34, 1.14)
Protestant		0.88 (0.78, 0.99)*		0.90 (0.71, 1.13)
Muslim		0.80 (0.72, 0.88)***		1.11 (0.90, 1.38)
Others		0.98 (0.64, 1.49)		1.12 (0.66, 1.92)
**Sex of household head (reference: male)**				

Female		1.08 (0.99, 1.17)		1.07 (0.94, 1.22)
**Reading newspaper (reference: No)**				
Yes		1.36 (1.26, 1.48)***		1.26 (1.11, 1.43)***
**Listening to radio (reference: No)**				
Yes		1.29 (1.20, 1.40)***		1.34 (1.19, 1.51)***
**Watching television (reference: No)**				
Yes		1.46 (1.33, 1.59)***		1.10, 0.96, 1.27)
**Marginalisationsex (sex & employment): Reference: unemployed female**				
Unemployed male		0.68 (0.57, 0.80)***		0.64 (0.45, 0.91)*
Employed female		1.03 (0.94, 1.12)		0.97 (0.85, 1.11)
Employed male		0.81 (0.74, 0.89)***		0.85 (0.73, 0.98)*
**Comprehensive knowledge of HIV/AIDS** (reference: No)				
Yes		1.16 (1.08, 1.24)***		1.17 (1.04, 1.30)**
**Accepting attitude** (reference: No)				
Yes		1.43 (1.33, 1.54)***		1.18 (1.05, 1.31)**
**Region (reference: Addis Ababa)**				
Tigray			1.58 (1.24, 2.02)***	1.68 (1.34, 2.12)***
Afar			1.55 (1.18, 2.04)**	1.52 (1.13, 2.04)**
Amhara			1.11 (0.87, 1.42)	1.16 (0.94, 1.44)
Oromia			0.66 (0.51, 0.85)**	0.59 (0.45, 0.78)***
Somali			0.47 (0.36, 0.63)***	0.52 (0.37, 0.74)***
Benshangul-Gumuz			1.20 (0.91, 1.57)	1.32 (1.004, 1.76)*
SNNPR			0.79 (0.61, 1.02)	0.88 (0.67, 1.14)
Gambela			1.65 (1.27, 2.14)***	1.87 (1.45, 2.41)***
Harari			0.75 (0.58, 0.97)*	0.68 (0.53, 0.87)**
Dire Dawa			1.52 (1.19, 1.95)**	1.58 (1.27, 1.96)***
**Marginalisation(wealth index, education, residence)**				

**reference:** Poor uneducated rural (PUR)				
Poor uneducated urban (PUU)			2.82 (1.60, 4.97)***	3.34 (1.01, 11.12)*
Poor educated rural (PER)			1.74 (1.52, 1.99)***	1.80 (1.45, 2.22)***
Poor educated urban (PEU)			2.70 (1.70, 4.29)***	2.22 (0.86, 5.78)
Rich uneducated rural (RUR)			2.12 (1.82, 2.47)***	1.86 (1.51, 2.30)***
Rich educated rural (RER)			2.68 (2.34, 3.06)***	2.67 (2.15, 3.31)***
Rich uneducated urban (RUU)			5.36 (4.36, 6.59)***	4.84 (3.59, 6.54)***
Rich educated urban (REU)			5.71 (4.84, 6.74)***	4.69 (3.60, 6.10)***
Variance (SE)	0.013 (1.3%)	0.011 (1.1%)	0.007 (0.7%)	0.011 (1.1%)
ICC %	0.214 (21.4%)	0.140 (14.0%)	0.077 (7.7%)	0.092 (9.2%)
**Model fit statistics**				
AIC	26702.48	25461.76	26096.69	22797.14
BIC	26718.77	25649.17	26251.5	23123.07
Log likelihood				-11358.57
**Standard logistic regression**				
AIC				23578.38
BIC				23896.16
Log likelihood				-11750.19
