# Supplementary material for: Intersectional inequity in knowledge, attitude, and testing related to HIV in Ethiopia: People with multiple disadvantages are left behind
Source: PLOS Glob Public Health. 2024 Aug 22;4(8):e0003628. doi: 10.1371/journal.pgph.0003628 (PMC11340895; doi:10.1371/journal.pgph.0003628)
Supplement: Attachment — Submitted filename: RESPON~1.DOC [file pgph.0003628.s003.DOC]

**PGPH-D-24-00849
Intersectional inequity in knowledge, attitude, and testing related to HIV in Ethiopia: People with multiple disadvantages are left behind**

**Dear Editor and Reviewers,**

We acknowledge your contribution in managing and evaluating our manuscript for publication. Your crucial comments and recommendations have helped enhance the quality of our work. In response to the reviewers’ feedback, we have addressed each comment and made necessary modifications. We have attached a response to the reviewers’ letter, outlining how we incorporated their suggestions into our revised manuscript. The reviewers’ suggestions and concerns are numbered, while our responses are in bold, underlined, and italic format.

**Editor Comments**:

The reviewers have provided some constructive feedback to strengthen the narrative of your manuscript. One suggestion revolves about additional analysis which may be helpful to consider as well, if possible. If such data are not available, do justify the approach in your revision.

***Authors’ response: Thank you very much for managing the review process for our manuscript. We truly appreciate the valuable feedback provided by the reviewers, which significantly enhanced the quality of our work. Regarding the suggestion related to additional analysis, the reviewer acknowledged that addressing it using the available DHS data was not feasible because all variables were not collected by DHS. Consequently, we agree with the reviewer’s concern and have included it as a recommendation for future researchers. Importantly, the findings from our analysis remain unaffected by the absence of the requested new analysis.***

**Reviewer's Responses to Questions**

**Comments to the Author**

1. Does this manuscript meet PLOS Global Public Health’s [publication criteria](https://journals.plos.org/globalpublichealth/s/criteria-for-publication)? Is the manuscript technically sound, and do the data support the conclusions? The manuscript must describe methodologically and ethically rigorous research with conclusions that are appropriately drawn based on the data presented.

Reviewer #1: Yes

Reviewer #2: Yes

***Authors’ response: Thank you so much for critically evaluating the manuscript.***

2. Has the statistical analysis been performed appropriately and rigorously?

Reviewer #1: Yes

Reviewer #2: Yes

***Authors’ response: Thank you so much for recognizing our work.***

3. Have the authors made all data underlying the findings in their manuscript fully available (please refer to the Data Availability Statement at the start of the manuscript PDF file)?

Reviewer #1: Yes

Reviewer #2: Yes

***Authors’ response: Thank you so much for reviewing the findings.***

4. Is the manuscript presented in an intelligible fashion and written in standard English?

PLOS Global Public Health does not copyedit accepted manuscripts, so the language in submitted articles must be clear, correct, and unambiguous. Any typographical or grammatical errors should be corrected at revision, so please note any specific errors here.

Reviewer #1: Yes

Reviewer #2: Yes

***Authors’ response: Thank you so much for reviewing the manuscript.***

**Review Comments to the Author**

1. Reviewer #1: This is a very good paper that documents people who have not accessed different HIV prevention and control messages and HIV testing services.

***Authors’ response: We sincerely appreciate your recognition of the objective and purpose of our work.***
2. I have two major comments:
• The global direction for HIV prevention, control, and treatment has shifted to key and priority populations due to resource constraints. This paper, however, covers the general population. Can you add one variable lumping key and priority populations together and compare it with the rest of the population with regard to the ‘triple disadvantages’? You may not find all key and priority populations in the DHS data but you can use whatever is available like divorced, widowed, separated, military/uniformed people, waitresses (may be as a proxy for female sex workers), daily laborers, factory workers, university students, etc. Such analysis may highlight the gap for policy makers.

***Authors’ response: The authors raised this concern during variable categorization. We appreciate your suggestions, particularly regarding the marginalized key and priority populations. Among these populations, we included the category of widowed or separated individuals in our analysis, comparing it with single or never-in-union individuals. Unfortunately, for other variables, we were unable to obtain data from DHS sources, as you previously mentioned. Nevertheless, we have included a recommendation statement for future researchers as follows:***

**The UNDP highlights intersectional determinants, including geography, discrimination, poverty, race, and socioeconomic status. However, some of these factors were not addressed in the current study. Therefore, it is crucial for future researchers to explore intersectionality from multiple dimensions. For instance, examining the intersection of female widows, sex workers, daily labourers, factory workers, long-distance travellers, and other vulnerable groups in the context of fighting HIV/AIDS is essential, as these individuals face a high risk of HIV infection.**
3. You used the term ‘Marginalisation based on wealth index, education, residence status’. Marginalization is a very strong word to use in this context since no one is deliberately going out of their way to discriminate against them, so they don’t access these services. They have just not been served for various reasons.

***Authors’ response: We greatly appreciate your valuable insight. In the revised submission, we have also taken your feedback into account and minimized the use of the term ‘Marginalization’.***
Minor comment:

4. Since you are discussing about inequality, it would have been easier to calculate and interpret odds ratio using rich, urban, educated as reference.

***Authors’ response: The primary objective of our research is to quantitatively address intersectional inequity using multiple (dis)advantage categories. As outlined in the method section, the triple (dis)advantage categories are established based on wealth status, residence, and education status. During the multilevel analysis, we observed that each individual variable (residence, wealth status, and education status) exhibits multicollinearity effects in relation to the triple (dis)advantage category. Consequently, we excluded residence, wealth status, and education status from the analysis, focusing solely on the triple categories, marital status, and other variable, as presented in Table 2.***

Reviewer #2:

1. A well written manuscript on an important topic. The authors have used a sound methodological approach to investigate the intersectional inequality issues in relation to HIV in Ethiopia. I have a few minor comments that the authors should consider making it a stronger narrative.

***Authors’ response: Thank you for the appreciation and reviewing our manuscript.***

2. The authors need to add some more narrative about stigma and anti-stigma approach in the introduction and some reflection about this in the discussion sections. Stigma intensifies existing power inequalities and social inequities, profoundly affecting various identities and health outcomes, especially in low and middle-income nations. You can read and include a recently published article on this topic by Majeed et al: Anti-stigma interventions in low-income and middle-income countries: a systematic review: <https://pubmed.ncbi.nlm.nih.gov/38707913/>

***Authors’ response: We have included this concept in the introduction section of the revised submission.*** **Attitudes towards people living with HIV manifest as stigma and discrimination. Previous studies investigated stigma and discrimination using similar questions related to attitudes towards people living with HIV. This issue remains a continuous concern in implementing prevention and control strategies for HIV/AIDS.**

***We have also added the following points in the discussion section of the revised submission:* The interventions aimed at enhancing knowledge about HIV/AIDS, ending stigma and discrimination, and expanding HIV testing coverage are also essential in addressing intersectional inequity. It is essential to adopt culturally sensitive and contextually relevant interventions. These might involve training initiatives and research activities, especially in low- and middle-income countries.**

3. In the discussion section, I feel that authors should reflect on the triple disadvantages and intersectionality and their close association with UNDP's multidimentional poverty index ([https://hdr.undp.org/content/2023-global-multidimensional-poverty-index-mpi#/indicies/MPI](https://hdr.undp.org/content/2023-global-multidimensional-poverty-index-mpi#/indicies/MPI)) or other indices of inequality such as the Human Development Index.

***Authors’ response: We have explained it in the discussion section of the revised submission as follows:* Improving the living conditions of communities is essential, especially by addressing multidimensional poverty, enhancing education, and tackling inequalities. Low-income adults face various disadvantages across multiple dimensions, with poverty significantly intersecting with health, education, and overall living conditions. The United Nations Development Programme (UNDP) emphasizes the need for countries to prioritize improving living conditions to promote a healthier population. Notably, a report covering 110 countries reveals that 25 of them successfully halved multidimensional poverty within 15 years. Despite this progress, 17.0% (1.1 billion out of 6.1 billion people) still live in poverty. Of these 1.1 billion individuals, 534 million reside in sub-Saharan Africa. The UNDP further underscores the importance of examining why people are left behind, empowering them, and implementing policies and reforms to address the drivers of poverty.**
